# Desaturation during exercise is not a sufficient mechanism for prediction of osteoporosis in non-cystic fibrosis bronchiectasis

**DOI:** 10.1186/s12890-020-1055-8

**Published:** 2020-01-28

**Authors:** Owen W. Tomlinson, Dimitris Vlachopoulos

**Affiliations:** Sport and Health Science, College of Life and Environmental Science, University of Exeter, St Luke’s Campus, Heavitree Road, Exeter, Devon EX1 2LU UK

**Keywords:** Desaturation, Pulmonary disease, Bone mineral density, Physical activity, Body composition

## Abstract

**Background:**

Recent research has proposed an association between desaturation during a six minute walking test (6MWT) and osteoporosis in an elderly group of individuals with non-cystic fibrosis bronchiectasis. A causative pathway through activation of hypoxia-inducible factor 1-alpha (HIF-1α) has been proposed.

**Commentary:**

Queries regarding the statistical approaches used are identified and discussed within this correspondence. These predominate around the use of linear regression models to predict osteoporosis in a group that is already osteoporotic, presenting with extreme values for bone mineral density (BMD). Further queries are raised regarding the HIF-1α pathway, and physical activity (PA) is proposed as an upstream mechanism for both reduced exercise tolerance and low BMD.

**Conclusions:**

It is suggested that osteoporosis cannot be predicted in a group that is already osteoporotic, and that PA is likely to be the causative mechanism between desaturation in the 6MWT and low BMD in non-cystic fibrosis bronchiectasis.

## Background

Previous research presented by the group of Huang et al. [1] has described the association between bone mineral density (BMD) in patients with non-cystic fibrosis bronchiectasis, and their respective oxygen desaturation during a six-minute walk test (6MWT). The authors concluded that *‘desaturation during 6MWT was a strong independent predictive factor for osteoporosis’* and that ‘*future research is warranted to clarify the underlying pathogenesis’*. However, there are areas of investigation within this study we believe warrant debate, particularly with regards to a) the collection and processing of variables of interest in this study to predict osteoporosis, and b) the potential mechanisms of bone development and maintenance in this disease group.

## Commentary

Firstly, we address concerns relating to the design of the regression model utilised for this study to predict osteoporosis. Whilst the authors report several factors predict for the presence of osteoporosis, for this claim to be made, authors would have needed to include ‘osteoporosis’ as a dichotomous categorical dependent variable within their regression model (as it is not a continuous variable). This approach would necessitate a logistic regression to identify the associated odds ratios of being, or not being, osteoporotic. The authors have instead utilised a linear regression to predict BMD, not osteoporosis. Furthermore, with regards to the BMD values themselves, participants present with a range of scores, depending on the skeletal site, with the majority (70%) of patients already having osteoporosis and a further 23% being osteopenic and therefore 93% of this sample have an abnormal BMD. This clearly indicates that desaturation during 6MWT cannot predict osteoporosis if the patients investigated *already* had osteoporosis. Therefore, the present study cannot claim that desaturation during 6MWT is a strong independent predictor of osteoporosis because this is misleading information and not supported by the design and the participants included in the study. This raises concerns about the selection bias of the present study as all the participants included had already osteopenia or osteoporosis.

In addition to concerns regarding statistical techniques used, there are concerns regarding the variables collated in this study. Approximately 15% of participants appear to present with very low BMD T-scores (< − 4),and given that a T-score of just − 2.5 is indicative of osteoporosis and according to relevant reference BMD values in female Chinese adults of the same age [2], the participants of present study had BMD T-scores as low as − 7 is highly unlikely and possibly a false result. If such proportion and values are true, they are very likely to bias final regression models. The authors should provide the actual BMD values in g/cm^2^ to allow appropriate comparison of the extremely low BMD T-scores reported.

Furthermore, the categorisation of being a ‘desaturator’ does not appear to be consistent with previous research. The authors of the present study cite Waatevik et al. [3], who in turn categorised desaturation as a change of ≥4% between pre- and post-test, and a post-test SpO_2_ < 90%. This is markedly different to the present study that used a decrease of 10%, or nadir SpO_2_ < 88%. If Huang et al [1] had indeed followed the previous research they cite, it is likely a larger number of patients would fit into their ‘desaturators’ category, skewing their sample distribution and changing the results of their regression models.

As a further point of note, we must query safety guidelines for desaturation in this study, as it would appear a number of individuals reached a nadir SpO_2_ < 80%, whereas international cardiopulmonary exercise testing guidelines [4] suggest this level of desaturation is a critical indicator for exercise termination, and has been correctly adhered to in previous studies [5]. Such extreme values of low SpO_2_ (whether in violation of good clinical practice, or not) could further bias both univariate and multivariate regressions by having a skewed distribution within models.

Consequently, of the variables associated with desaturation, only being categorised as a ‘desaturator’ itself presents as a significant predictor in the final regression model. However, it is not clear how this final model has been biased by the above points of discussion and we therefore query whether desaturation during exercise is really a true predictor for ‘osteoporosis’ (or BMD below a T-score of − 2.5 in actuality). When the aforementioned queries are considered alongside previous research in idiopathic pulmonary fibrosis (which also pathologically presents with impaired pulmonary function) that have shown a weak association between nadir SpO_2_ in a 6MWT and BMD [6], we believe the authors have inadvertently identified a predictive association where one does not exist. However, if desaturation during exercise was a true and legitimate predictor when considering the above points, then it is only a predictor for a low BMD T-score in a group that is *already* osteoporotic, and not the presence of, nor risk of developing, osteoporosis itself.

Secondly, in addition to aforementioned concern regarding data, there are concerns relating to proposed mechanisms described by authors. Despite concluding that the underlying pathogenesis requires further research, the authors instead describe in detail a pathway that could account for the observed reduction in BMD – the stimulation of hypoxia-inducible factor 1-alpha (HIF-1α). However, their cited review of Maes et al. [7], suggests that HIF-1α is only activated when O_2_ drops < 5% (a large difference to the lowest reported nadir SpO_2_ of ~ 60%); although the relative definitions of ‘hypoxia’ and ‘normoxia’ can vary dependent upon organ systems in question. This discrepancy immediately calls the validity of this pathway into question.

To further this, it is unclear from the study how desaturation was associated with 6MWT performance or exercise intensity, and therefore we cannot identify with confidence a) how often patients with non-cystic fibrosis bronchiectasis may experience acute episodes of desaturation during daily living, and b) how severe these hypoxic episodes would have to be to result in cumulative or permanent hypoxic status, which would be required to stimulate the HIF-1α pathways proposed by the authors. In contrast to the proposed HIF-1α mechanisms of Huang et al. [1], we suggest that physical activity (PA) is instead a contributory factor, for multiple reasons.

It is well established that PA plays a crucial role for BMD in patients with osteopenia and osteoporosis and especially for postmenopausal women that were part of the current study [8]. PA can affect BMD [9] and physical fitness, such as 6MWT performance [10] of postmenopausal women and elderly men and directly affects two of the main outcomes (BMD and 6MWT) in the present study, but it was not appropriately measured or controlled. This raises concerns about the reliability of the findings considering that other important factors, such as PA, affect the primary outcomes but not controlled.

Furthermore, it should be noted how PA affects body composition, and how lean body mass may affect BMD in the population examined. There is conclusive evidence regarding lean mass being one of the primary determinants of BMD across the lifespan and specifically in elderly population due to the increased prevalence of sarcopenia [11]. Lean mass is affected by PA activity levels and acts directly or indirectly on BMD in older men and women indicating that it should be taken into account [12]. The 6MWT performance also depends on lean mass [13], but the present study did not include the lean mass of the patients investigated, either as an absolute value or as a percentage of total mass, and did not adjust the relationships provided for the lean mass status which is a major limitation considering the crucial role of lean mass for BMD in patients with bronchiectasis [14].

In addition to the above mechanistic factors, recent research that has shown PA is low in patients with non-cystic fibrosis bronchiectasis relative to non-diseased controls; and positively associated with measures of lung function and exercise performance [15]. Given these factors, we believe that it is more likely that reduced PA is responsible for low BMD in this population, and not the HIF-1α pathway, although we admittedly do not have evidence to completely refute the HIF-1α pathway. The authors of this study do acknowledge that PA was not measured during this study the analyses are retrospective in nature and therefore the authors will be limited by number of confounding variables available to them for analyses. Given the relative ease with which basic subjective measures of PA can be obtained (e.g. questionnaires), future prospective analyses should seek to include some measure of PA in order to provide insight into these proposed mechanisms.

## Conclusion

In summary, whilst the work of Huang et al. [1] is well intentioned, by identifying the importance of exercise performance as an important clinical factor to consider in non-cystic fibrosis bronchiectasis, we respectfully disagree with methods and findings, and interpret these results differently. Consequently, we believe that a) the authors are not predicting ‘osteoporosis’ as they claim, only reduced BMD in an already osteoporotic group, and b) a failure to acknowledge PA as an upstream mechanism that simultaneously accounts for both low BMD and desaturation during exercise has led authors to inferring a causative effect between two independent variables (BMD and SpO_2_) where one does not exist.

## Data Availability

Not applicable.

## Abbreviations

6MWT: six minute walk test
BMD: Bone mineral density
HIF-1 α: Hypoxia-inducible factor 1-alpha
PA: Physical activity
